# Sol-Gel Synthesis, Structure, Morphology and Magnetic Properties of Ni_0.6_Mn_0.4_Fe_2_O_4_ Nanoparticles Embedded in SiO_2_ Matrix

**DOI:** 10.3390/nano11123455

**Published:** 2021-12-20

**Authors:** Thomas Dippong, Erika Andrea Levei, Iosif Grigore Deac, Ioan Petean, Gheorghe Borodi, Oana Cadar

**Affiliations:** 1 Faculty of Science, Technical University of Cluj-Napoca, 76 Victoriei Street, 430122 Baia Mare, Romania; 2 INCDO-INOE 2000, Research Institute for Analytical Instrumentation, 67 Donath Street, 400293 Cluj-Napoca, Romania; erika.levei@icia.ro (E.A.L.); oana.cadar@icia.ro (O.C.); 3 Faculty of Physics, Babes-Bolyai University, 1 Kogalniceanu Street, 400084 Cluj-Napoca, Romania; iosif.deac@phys.ubbcluj.ro; 4 Faculty of Chemistry and Chemical Engineering, Babes-Bolyai University, 11 Arany Janos Street, 400028 Cluj-Napoca, Romania; ioan.petean@ubbcluj.ro; 5 National Institute for Research and Development of Isotopic and Molecular Technologies, 65-103 Donath Street, 400293 Cluj-Napoca, Romania; borodi@itim-cj.ro

**Keywords:** zinc-manganese ferrite, sol-gel, nanocomposite, magnetic properties

## Abstract

The structure, morphology and magnetic properties of (Ni_0.6_Mn_0.4_Fe_2_O_4_)_α_(SiO_2_)_100−α_ (α = 0–100%) nanocomposites (NCs) produced by sol-gel synthesis were investigated using X-ray diffraction (XRD), Fourier transform infrared spectroscopy (FT-IR), atomic force microscopy (AFM) and vibrating sample magnetometry (VSM). At low calcination temperatures (300 °C), poorly crystallized Ni_0.6_Mn_0.4_Fe_2_O_4_, while at high calcination temperatures, well-crystallized Ni_0.6_Mn_0.4_Fe_2_O_4_ was obtained along with α-Fe_2_O_3_, quartz, cristobalite or iron silicate secondary phase, depending on the Ni_0.6_Mn_0.4_Fe_2_O_4_ content in the NCs. The average crystallite size increases from 2.6 to 74.5 nm with the increase of calcination temperature and ferrite content embedded in the SiO_2_ matrix. The saturation magnetization (*Ms*) enhances from 2.5 to 80.5 emu/g, the remanent magnetization (*M_R_*) from 0.68 to 12.6 emu/g and the coercive field (*H_C_)* from 126 to 260 Oe with increasing of Ni_0.6_Mn_0.4_Fe_2_O_4_ content in the NCs. The SiO_2_ matrix has a diamagnetic behavior with a minor ferromagnetic fraction, Ni_0.6_Mn_0.4_Fe_2_O_4_ embedded in SiO_2_ matrix displays superparamagnetic behavior, while unembedded Ni_0.6_Mn_0.4_Fe_2_O_4_ has a high-quality ferromagnetic behavior.

## 1. Introduction

Nanosized mixed metal oxides with high surface area and small particle size display unique properties [1]. MFe_2_O_4_ (M = Zn, Co, Mn, Ni, etc.) type magnetic spinel ferrites with the general formula have numerous applications due to their high reactivity, chemical stability, optical, electrical and catalytic/ photocatalytic behaviors. Additionally, this type of magnetic nanoparticle is easily separated and recycled without important loss of their chemical activity [1,2].

Nickel ferrite (NiFe_2_O_4_) has an inverse spinel structure with Ni^2+^ ions occupying octahedral (B) sites and Fe^3+^ ions occupying tetrahedral (A) as well as octahedral (B) sites. It presents high saturation magnetization (*M_S_*), resistivity and low losses over a large frequency range, that resulted in applications in diverse fields [3,4]. Manganese ferrite (MnFe_2_O_4_) is of great interest due to its good biocompatibility, coloristic properties, tunable magnetic properties, guidability in a magnetic field and excellent chemical stability. MnFe_2_O_4_ nanoparticles are also recognized as efficient agents for magnetic hyperthermia and magnetic resonance imaging [1,2,3,4,5]. MnFe_2_O_4_ has a spinel crystal structure with Fe^3+^ ions occupying the octahedral sites and Mn^2+^ ions occupying the tetrahedral sites [4]. At calcination temperatures above 900 °C, a part of the Mn^2+^ ions migrate from tetrahedral (A) to octahedral (B) sites leading to a mixed spinel structure [4,6]. Both pure and doped MnFe_2_O_4_ tend to form anti-ferromagnetic α-Fe_2_O_3_ phase when are thermally treated at 200 °C in open air, but at higher calcination temperatures, the anti-ferromagnetic α-Fe_2_O_3_ phase is no longer remarked [7].

The substitution of NiFe_2_O_4_ with magnetic divalent transition metal ions like Mn^2+^ received considerable interest due to appealing magnetic and electrical features. Mixed Ni-Mn ferrites are frequently used, as besides the good magnetic properties, they also have large resistivity, permeability and small losses in comparison with other dielectrics [3,6]. Ni-Mn ferrites show interesting magnetic properties which recommend them to be used as hard or soft magnets and for high-frequency applications. The ferrite structure and magnetic properties are sensitive to synthesis methods, additive substitutions and calcination process [8]. By adjusting the Mn to Ni ratio in the ferrite, the magnetic properties of the ferrite can be controlled [3]. By substitution of Mn^2+^ ions with Ni^2+^ ions, Ni^2+^ ions occupy octahedral (B) sites, while Mn^2+^ ions are distributed between tetrahedral (A) and octahedral (B) sites [7].

The particle size and shape have a critical role in determining the ferrite magnetic characteristics [7]. Nanosized magnetic materials have a so-called critical particle size below which the crossover from a single- to a multidomain structure is possible. In single-domain systems, below the so-named blocking temperature, the magnetic anisotropy governs the spin alignment along the magnetization axis [7]. The presence of Mn^2+^ ions in Ni ferrites changes their structural, magnetic, electrical and dielectric properties [9]. Surface spins, spin canting and reduction of particle sizes also influence the magnetic properties [8].

The wide-scale applications of nanosized ferrites boosted the development of numerous synthesis methods. Generally, the spinel ferrites are synthesized by the ceramic technique which involves high temperatures and produces particles with low specific surface area. Alternative synthesis methods are co-precipitation, sol-gel, hydrothermal, micro-emulsion, heterogeneous precipitation, sonochemistry, solid-state, combustion, etc. [1,2,3,4]. Generally, the chemical methods allow the production of fine-grained ferrites, but requests a long reaction time and post-synthesis thermal treatment, and produces ferrites with poor crystallinity and broad particle size distribution [1]. Recently, the development of low-cost synthesis methods that allow the production of nano-sized, single-crystalline and single-phase powders has become of great interest [4].

The sol-gel route is an easy way to produce ferrite-based NCs (nanocomposites) as it is a simple low-cost process and concedes the control of structure and properties [5]. The sol-gel method allows the production of nanosized composite materials containing highly dispersed magnetic ferrite particles [9]. For a better control of the particle size and particle agglomeration reduction, the coating of ferrite with silica (SiO_2_) is often used. The SiO_2_ coating also improves the magnetic properties and biocompatibility of the ferrites due to its bio-inert behavior in contact with living tissue [5]. Most of the organic surfactants reduce the biocompatibility due to their inflammatory reactions. Oppositely, the SiO_2_ is bioinert and a widely accepted material by the living body, the SiO_2_ coating of ferrite nanoparticles preventing the direct contact with the living tissue and diminishing the possible inflammatory risk. Moreover, the organic surfactant layer can be removed from the nanoparticles in contact with the living tissue, revealing the ferrite surface. The SiO_2_ layer cannot be solved or removed by the living tissue maintaining the optimal biocompatibility of the ferrite nanoparticles [10]. Tetraethyl orthosilicate (TEOS) is a network forming agent commonly used in the sol-gel synthesis, because it forms strong networks with moderate reactivity, permits the incorporation of various organic and inorganic molecules and offers short gelation time [5,11].

This study investigates the influence of the mixed Ni-Mn ferrite embedding in various contents of amorphous SiO_2_ matrix, at different calcination temperatures on the structure, morphology and magnetic properties of (Ni_0.6_Mn_04_Fe_2_O_4_)_α_(SiO_2_)_100−α_ NCs using X-ray diffraction (XRD), Fourier transform infrared spectroscopy (FT-IR), atomic force microscopy (AFM) and vibrating sample magnetometry (VSM).

## 2. Materials and Methods

(Ni_0.6_Mn_0.4_Fe_2_O_4_)_α_(SiO_2_)_100−α_ (α = 0–100%) NCs were obtained by the sol-gel method. Nickel nitrate (Ni(NO_3_)_2_·6H_2_O), manganese nitrate (Mn(NO_3_)_2_·3H_2_O) and ferric nitrate (Fe(NO_3_)_3_·9H_2_O) were dissolved in 1,4-butanediol (1,4BD) in a molar ratio of 0.6:0.4:2:8. TEOS dissolved in ethanol and acidified with nitric acid (pH = 2) was added to the nitrate-1,4BD mixture, under continuous stirring, at room temperature, using an NO_3_^−^:TEOS molar ratio of 0:2 (α = 0%), 0.5:1.5 (α = 25%), 1:1 (α = 50%), 1.5:0.5 (α = 75%) and 2:0 (α = 100%). The obtained sol was left in open air for gelation; afterwards, the solid gels were grinded and calcined in air, at 300, 700 and 1100 °C for 5 h using an LT9 muffle furnace (Nabertherm, Lilienthal, Germany).

The structure of NCs was investigated by XRD using a D8 Advance (Bruker, Karlsruhe, Germany) diffractometer, operating at 40 kV and 40 mA and employing CuKα radiation with λ = 1.54060 Å, at room temperature. The formation of the ferrite and SiO_2_ matrix were investigated using a Spectrum BX II (Perkin Elmer, Waltham, MA, USA) Fourier-transform infrared spectrometer in the range of 400–4000 cm^−1^. AFM was carried-out using a JSPM 4210 (JEOL, Tokio, Japan) scanning probe microscope using NSC15 silicon nitride cantilevers with resonant frequency of 325 kHz and force constant of 40 N/m, in tapping mode. The NCs were dispersed into ultrapure water, transferred on glass slides by vertical adsorption for 30 s and dried in air. Several areas of variable size (2.5 µm × 2.5 µm to 1 µm × 1 µm) of the dried glass slides were scanned. A cryogenic VSM magnetometer (Cryogenic Ltd., London, UK) was used for the magnetic measurements. The *M_S_* was determined in high magnetic field up to 10 T, whereas the magnetic hysteresis loops were conducted on samples incorporated in an epoxy resin to avoid any particle movement, between −2 to 2 T, at 300 K.

## 3. Results and Discussion

**Figure 1:**
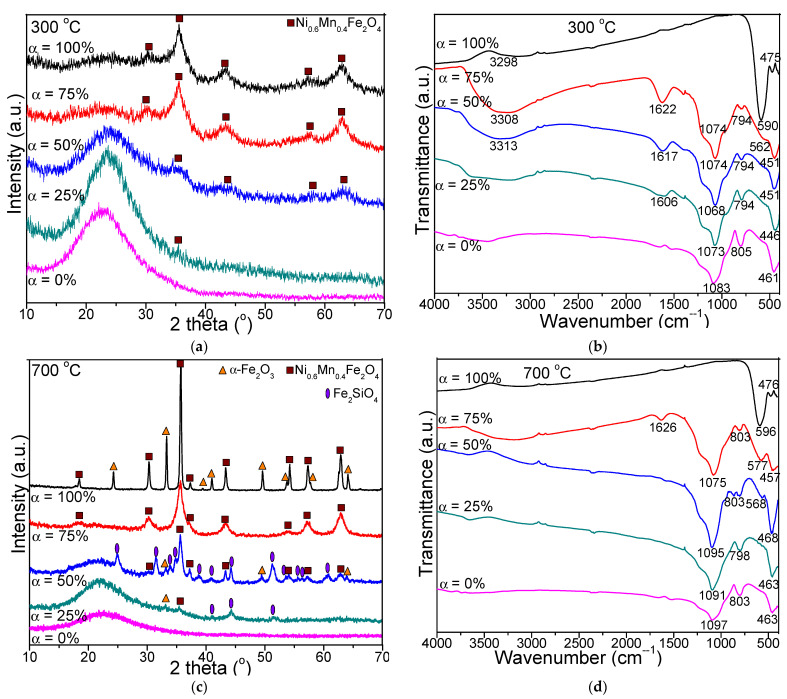

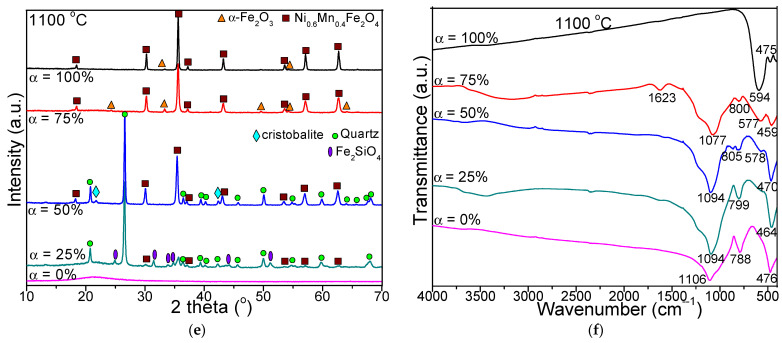
XRD patterns (**a**,**c**,**e**) and FT-IR spectra (**b**,**d**,**f**) of (Ni_0.6_Mn_04_Fe_2_O_4_)_α_(SiO_2_)_100__−__α_ (α = 0–100%) NCs calcined at 300, 700, 1100 °C.

The XRD patterns and FT-IR spectra of the (Ni_0.6_Mn_0.4_Fe_2_O_4_)_α_(SiO_2_)_100−α_ (α = 0, 25, 50, 75, 100%) NCs calcined at 300, 700 and 1100 °C are presented in Figure 1. At all calcination temperatures, in case of NCs with α = 0%, the formation of amorphous SiO_2_ matrix is supported by the broad halo located at 2θ = 15–30° in the XRD pattern. At 300 °C, the NC with α = 25% is amorphous, the nano-crystalline state developing by increasing the value of α (Figure 1a). In case of the NCs calcined at 700 and 1100 °C (Figure 1c,e), the observed peaks indicate the presence of the cubic spinel structure of Mn_x_Ni_1−x_Fe_2_O_4_. The MnFe_2_O_4_ (JCPDS card no. 74-2403) has a lattice parameter of 8.511 Å, while the NiFe_2_O_4_ (JCPDS card no. 10-0325) [12] has a lattice parameter of 8.339 Å. The Mn_x_Ni_1−x_Fe_2_O_4_ is isostructural with the two structures mentioned above, Ni and Mn being in the same position with an occupancy factor of x for Mn and 1−x for Ni. The reflection planes (220), (311), (222), (400), (422), (511) (440) and (533) belonging to the angular positions at 2θ = 29.99°, 35.33°, 36.83°, 42.87°, 53.11°, 56.65°, 62.16° and 73.38° are consistent with the spinel structure corresponding to the Fd3m space group and match with the literature data [13]. From the positions of diffraction lines for Mn_x_Ni_1−x_Fe_2_O_4_ result a lattice parameter of 8.44 Å. From the lattice parameter which has a linear dependence with x, results x = 0.6, and Ni_0.6_Mn_0.4_Fe_2_O_4_.

At 700 °C, in case of NC with α = 75%, the single and well-crystallized Ni_0.6_Mn_04_Fe_2_O_4_ is observed, while in the case of NC with α = 100%, the α-Fe_2_O_3_ (JCPDS card no. 89-0599 [12]) secondary phase is also present. The presence of α-Fe_2_O_3_ might be attributed to partially embedding of the ferrite in the SiO_2_ matrix, due to the low content or lack of SiO_2_ and the short time or calcination temperature required to produce pure crystalline Ni_0.6_Mn_04_Fe_2_O_4_ phase [5].

As the ferrite content decreases, in NCs with α = 25 and 50%, besides Ni_0.6_Mn_04_Fe_2_O_4_, the presence of α-Fe_2_O_3_ and Fe_2_SiO_4_ (JCPDS card no. 87-0315 [12]) secondary phases is also noticed. We assume that the formation of Fe_2_SiO_4_ could be related to difficulty of oxygen diffusion from the pores of SiO_2_ matrix and partial reduction of Fe^3+^ into Fe^2+^, which reacts with the SiO_2_ matrix and forms Fe_2_SiO_4_ under the reducing condition produced by the decomposition of carboxylate precursors.

At 1100 °C, in case of NCs with α = 75–100% the well-crystallized Ni_0.6_Mn_0.4_Fe_2_O_4_ phase together with traces of α-Fe_2_O_3_ secondary phase are observed. In NCs with α = 50% containing ferrite and SiO_2_ matrix in 1:1 molar ratio, besides the main phase of Ni_0.6_Mn_04_Fe_2_O_4_, the secondary phases of crystallized SiO_2_ matrix are also noticed (cristobalite, JCPDS card no. 89-8936 and quartz, JCPDS card no. 89-8936 [12]), while in NC with α = 25%, Fe_2_SiO_4_ is also obtained. Although it was reported that the thermal treatment may induce polymorphous transitions in Fe_2_O_3_, especially in the case of nanosized powders or nanoparticles embedded in amorphous and porous SiO_2_ matrix, in our case only α-Fe_2_O_3_ was observed [14]. The peaks corresponding to ferrite become more intense at 1100 °C, indicating high degree of crystallinity, crystallite size (due to the crystal coalescence process), nucleation rate and low effect of the inert surface layer [5]. Also, the highest peak shifts to higher angles with increasing Ni_0.6_Mn_04_Fe_2_O_4_ content embedded in the SiO_2_ matrix.

Among the available methods to estimate the crystallite size, those using the diffraction profile analysis, namely Williamson-Hall and Warren-Averbach procedures, require several diffraction profiles [15,16]. Considering that in our case, especially at low calcination temperatures, we have only few diffraction peaks, we estimated the average crystallite using the Scherrer method, which requires the full the width at half maximum (FWHM) for a single diffraction line [9]. Though the X-ray profile analysis is an average method, it is still a reliable method for measuring the crystallite size, apart from transmission electron microscopy (TEM). The average crystallite size of NCs calculated using the Debye-Scherrer formula [3,17] are presented in Table 1. The low ferrite content embedded in the amorphous SiO_2_ matrix retards the expansion of the crystallite size, whereas high ferrite content favors both nucleation and growth of crystallite size at the nucleation centers, leading to higher crystallite size [1]. By increasing the calcination temperature, the Ni^2+^ and Fe^3+^ ions tend to occupy specific positions in the crystal lattice of the ferrite [18,19]. The crystallites were more compact at low ferrite content embedding in SiO_2_, since the smaller Ni^2+^ ion can dissolve in the spinel lattice, while high ferrite content embedding in SiO_2_ matrix causes the increase of the porosity leading to higher crystallite size [18]. During the calcination process, coalescence occurs, the smaller crystallites being merged together to form the large crystallites [7].

At all temperatures, the FT-IR spectra (Figure 1b,d,f) of NCs with α = 25–100% show the absorption bands corresponding to the vibration of tetrahedral M–O (M=Ni, Mn) bonds at 568–596 cm^−^^1^ and of octahedral M–O (M=Fe) bonds at 446–476 cm^−^^1^ [1,3,4,17]. The different vibration frequencies of M–O groups are a consequence of the higher M–O bond length in octahedral (B) sites than that in tetrahedral (A) sites. The presence of these two absorption bands in FT-IR spectra confirms that the ferrites have cubic spinel structure. The intensity of the band at 568–596 cm^−^^1^ is larger than that of 446-476 cm^−^^1^, indicating that the vibration of tetrahedral M–O is higher than of octahedral M–O groups [3]. Generally, the Ni^2+^ ions occupy the octahedral (B) sites, whereas Mn^2+/^Fe^3+^ ions prefer both octahedral (B) and tetrahedral (A) sites [17]. The absorption bands shifting to lower values is accredited to the movement of Fe^3+^, Mn^2+^ and Ni^2+^ ions corresponding to the O^2−^ ions in the octahedral (B) and tetrahedral (A) sites, and consequently the change of the Fe^3+^–O^2−^(M^3+^–O^2−^) and M^2+^–O^2−^ bond length, respectively [4]. The intensity of the vibrational band at 568–596 cm^−^^1^ increases with the increasing calcination temperature, due to the increasing ferrite crystallinity, since the ferrites consist of crystals bonded to all adjacent neighbors through ionic, covalent or van der Waals forces [5,11,20,21].

The small shift of the vibrational band originates from the movement of ions among the tetrahedral (A) and octahedral (A) sites as a result of the increasing calcination temperature [5,11,21]. The characteristic bands of the SiO_2_ matrix were detected in the FT-IR spectra of NCs with α = 0–75%, as follows: 1068–1106 cm^−^^1^ with a shoulder at about 1200 cm^−^^1^ related to vibration of Si–O–Si chains, 788–805 cm^−^^1^ related to the vibrations of SiO_4_ tetrahedron and 446–476 cm^−^^1^ related to the Si–O bond vibration and overlapping the band of Fe–O vibration [5,11]. The high intensity of these bands indicates a low polycondensation degree of the SiO_2_ network [5]. The broad peaks observed at 3298-3313 cm^−^^1^ and at 1606–1626 cm^−^^1^ are ascribed to the vibrations of the –OH group and hydrogen bonds from adsorbed water molecules [1].

AFM was previously used to study the temperature effect on Ni and Mn ferrite nanoparticles transferred as thin film onto solid substrate. Ashiq et al. evidenced by AFM that Ni ferrite nanoparticles dispersion in liquid environment is proper to obtain well-structured thin films [22]. Moreover, Tong et al. reported particle diameters of 25 nm at 400 °C; 44 nm at 500 °C and 65 nm at 700 °C, and surface roughness depending on the nanoparticle disposal in the topography [23].

**Figure 2:**
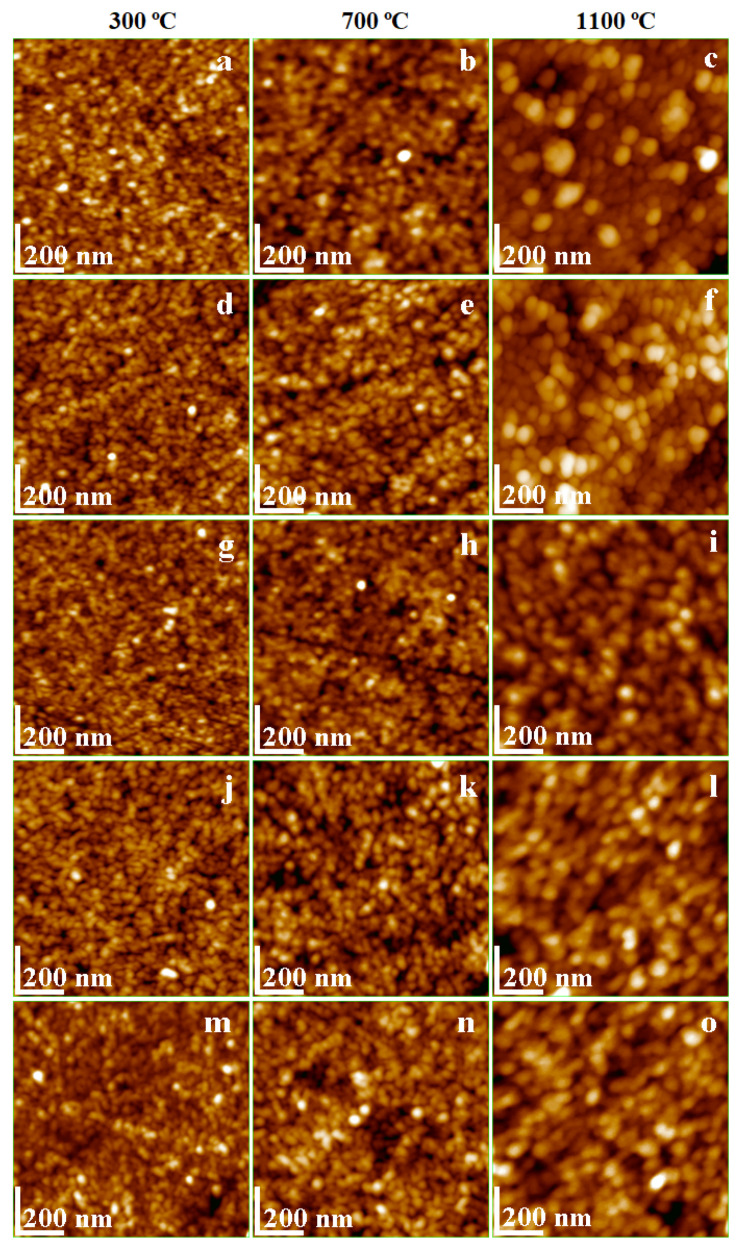
AFM topographic images of (Ni_0.6_Mn_0.4_Fe_2_O_4_)_α_(SiO_2_)_100−α_ NCs, α100% (**a**,**b**,**c**); α = 25% (**d**,**e**,**f**); α = 50% (**g**,**h**,**i**); α = 75% (**j**,**k**,**l**) and α = 100% (**m**,**n**,**o**) calcined at 300, 700 and 1100 °C.

The use of Mn ferrite nanoparticles as dispersed matter into the liquid environment as magnetic ink was also reported [24]. The printed thin film investigated with AFM revealed Mn ferrite nanoparticles of about 95 nm and the surface roughness depending on the particle diameter and on the observed agglomeration tendency [24]. The AFM topographic images are presented in Figure 2a–o. A dependence of nanoparticle diameter on the calcination temperature was observed for pure Ni_0.6_Mn_04_Fe_2_O_4_ (Figure 2a–c). The diameter of the round shape particles increases from about 18 nm at 300 °C to 52 nm at 700 °C, and 75 nm at 1100 °C, respectively. The crystallite size increase with the temperature increase was also observed based on the XRD data. The particle size revealed by AFM correlation with XRD crystallite size of pure Ni-Mn ferrite indicates a polycrystalline state at low temperatures (crystallite size is considerably smaller than particle size) and monocrystalline state (crystallite size is very close to the particle diameter) at 1100 °C. Establishing a certain number of crystallites per particle requires a more enhanced investigation based on scanning electron microscopy (SEM) and Brunauer–Emmett–Teller (BET) analysis [16].

XRD patterns show that the SiO_2_ matrix is amorphous at all calcination temperatures. However, the particle size and shape evolution with increasing temperature may be observed using AFM. Figure 2m reveals small round shape nanoparticles and a diameter increasing with the calcination temperature, i.e., about 12 nm at 300 °C, 28 nm at 700 °C and 35 nm at 1100 °C (Figure 2n,o). Previous studies confirm the shape and sizes of the silica nanoparticles observed by AFM [25,26].

The NCs with α = 25–75% combine the morphological and structural features of both Ni-Mn ferrite and amorphous SiO_2_ nanoparticles. The diameter of the round-shape nanoparticles is strongly influenced by the calcination temperature and composition (Figure 2d–l). The lowest size particles were observed at 300 °C and the bigger ones at 1100 °C (Table 1). The amorphous SiO_2_ matrix increases the particle size compared to the ferrite crystallites due to the embedding effect. This effect is more visible at 300 °C than at 1100 °C. At higher calcination temperatures, the ferrite crystallite is well covered by an amorphous SiO_2_ layer which forms the composite nanoparticle. The insulating behavior of the amorphous SiO_2_ matrix prevents the overgrowth of magnetic domains and guarantees the nano-structural stability. A slight decrease of the nanoparticle size occurs by increasing the amorphous SiO_2_ content. This decrease is most obvious at 1100 °C (Figure 2c,f,i,l), where the amorphous SiO_2_ matrix inhibits the development of bigger ferrite crystallites (Table 1). A similar behavior was reported for other ferrite systems [5,11].

**Figure 3:**
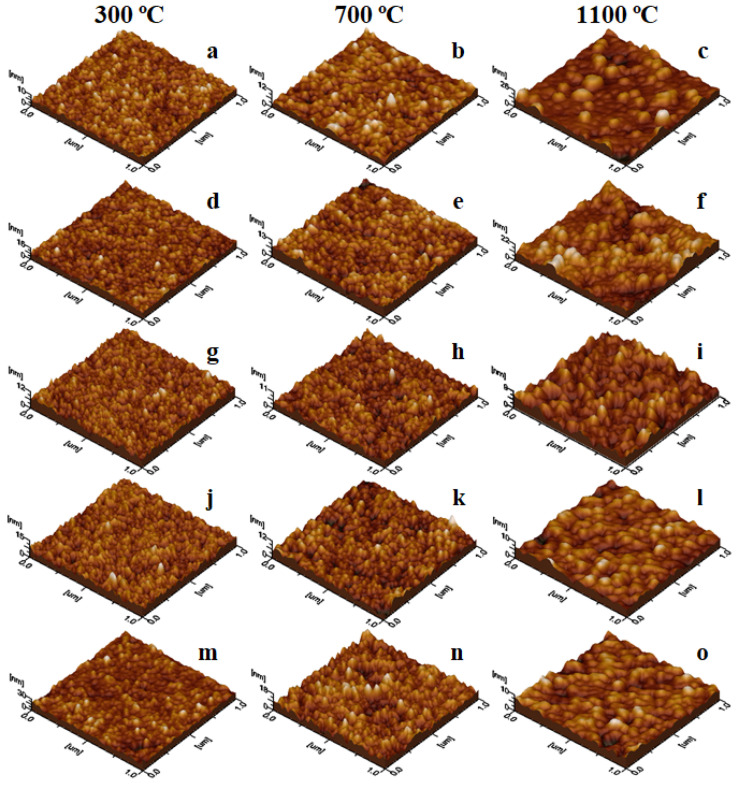
3D AFM images of (N_i0_._6_Mn_0.4_Fe_2_O_4_)_α_(SiO_2_)_100−α_ NCs α = 100% (**a**–**c**); α = 25% (**d**–**f**); α = 50% (**g**–**i**); α = 75% (**j**–**l**) and α = 100% (**m**–**o**) calcined at 300, 700 and 1100 °C.

The powder dispersion in an aqueous environment facilitates the nanoparticle arrangement, assuring a uniform adsorption onto the solid substrate creating well-structured thin films [27], as observed in Figure 3a–o. The film roughness depends on the nanoparticle diameter and their disposal on the substrate surface. Thus, the lower roughness values are obtained at 200 °C (Figure 3a,d,g,j,m) due to the uniform adsorption of fine nanoparticles. The particle diameter increases with the calcination temperature, while the adsorbed film uniformity depends on the local heights formed by bigger nanoparticles (Figure 3c,f,i,l).

The morphological aspects of the nanoparticle thin films revealed by AFM correlated with the magnetic properties allow the design of functionalized surfaces for various applications where thermal deposition at high temperatures it is not possible, i.e., such as polymer coating.

**Figure 4:**
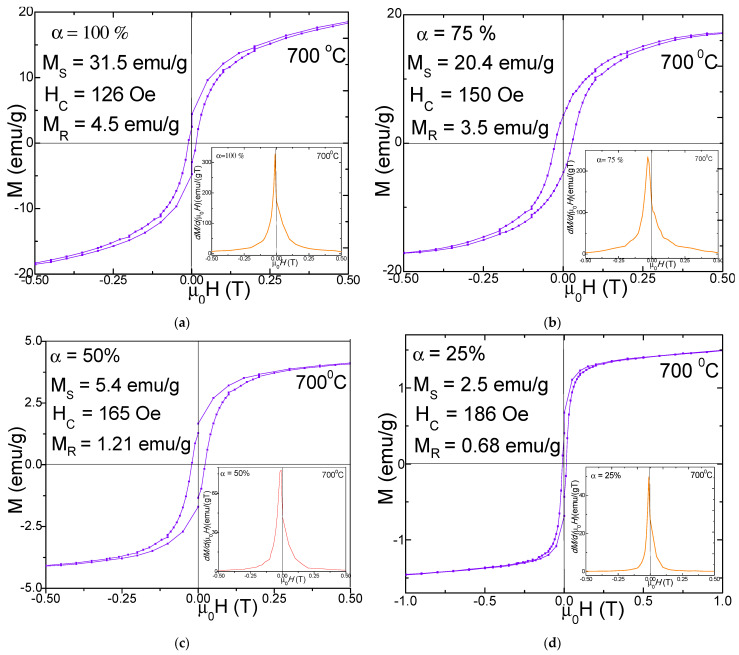
Magnetic hysteresis loop and magnetization derivative of (N_i0_._6_Mn_0.4_Fe_2_O_4_)_α_(SiO_2_)_100−α_ (α = 100% (**a**), 75% (**b**), 50% (**c**) and 25% (**d**)) NCs calcined at 700 °C.

**Figure 5:**
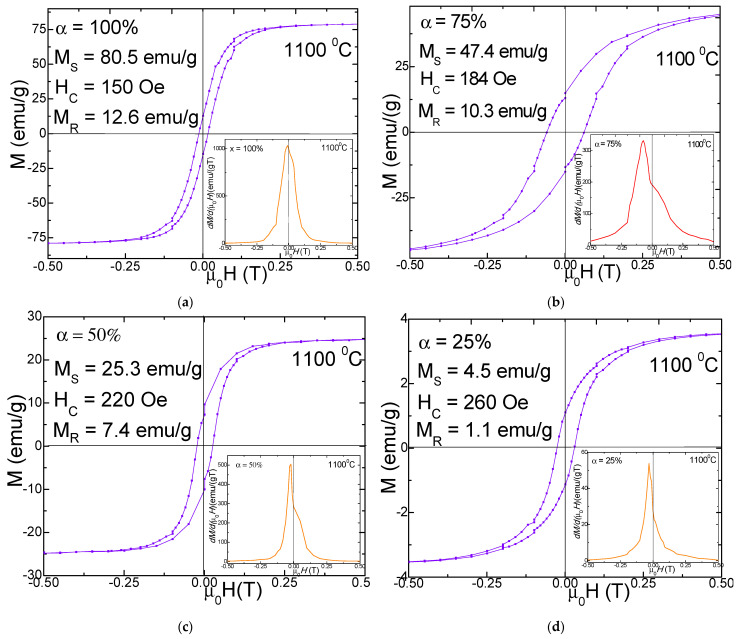
Magnetic hysteresis loop and magnetization derivative of (N_i0_._6_Mn_0.4_Fe_2_O_4_)_α_(SiO_2_)_100−α_ (α = 100% (**a**), 75% (**b**), 50% (**c**) and 25% (**d**)) NCs calcined at 1100 °C.

Figure 4 and Figure 5 display the magnetic hysteresis loops and *dM*/*d*(*µ*_0_*H*)) derivatives (in insets) as well as the saturation magnetization (*M_S_*), remnant magnetization (*M_R_*) and coercivity (*H_C_*) values for (N_i0_._6_Mn_0.4_Fe_2_O_4_)_α_(SiO_2_)_100−α_ (α = 25–100%) NCs calcined at 700 and 1100 °C. The hysteresis loops are very narrow, indicating that the nanoparticles have soft magnetic behavior. The derivatives of the hysteresis loops (total susceptibility) represent the local slope of M-H curves. A single sharp maximum in the *dM*/*d*(*µ*_0_*H*) vs. H curves suggests the presence of a single magnetic phase.

The peaks’ broadening indicates a larger distribution of the particle sizes. For the NCs with α = 25–100%, *dM/d*(*µ*_0_*H*) vs. H curves have a single and sharp peak. The morphology and the phase purity of NCs, as well as their magnetic properties, are strongly affected by the calcination temperature [3,5]. The SiO_2_ matrix has diamagnetic behavior for both 700 and 1100 °C calcination temperatures. For the NCs with α = 100% (Ni_0.6_Mn_0.4_Fe_2_O_4_), typical hysteresis loops for ferromagnetic materials were obtained, for all the calcination temperatures, due to the presence of larger size crystallites and particles as found in XRD and AFM analyses. The unembedded Ni_0.6_Mn_0.4_Fe_2_O_4_ (α = 100%) has a much higher *M_S_*, especially when it is calcined at 1100 °C, than the ferrites embedded in the SiO_2_ matrix (α = 25–75%), with pretty narrow hysteresis loops, close to a superparamagnetic behavior.

The superparamagnetic-like behavior of the NCs is a consequence of the low sizes of the crystallites and of their low magnetic anisotropy which allow their easily thermal activation [3,4]. The increase of the calcination temperature can lead to the improvement of *M_S_* and *M_R_*, as a result of a better crystallinity of the ferrite, of proper interatomic lengths changing of the atomic coordination number, etc. The *M_S_* values of NCs with high content of Ni_0.6_Mn_0.4_Fe_2_O_4_ embedded in the SiO_2_ matrix are larger due to larger particles sizes which show reduced spin canting and other surface effects which are usually present in small size particles. The main mechanisms of the magnetization process are the domain wall motions and the magnetic moment rotations [3]. The spin disorder on the nanoparticle surface can also strongly affect the *M_S_* value. Moreover, the lattice defects can weaken the magnetic super-exchange interaction between the tetrahedral (A) and the octahedral (B) sites [3]. The involved magnetic Fe^3+^, Ni^2+^ and Mn^2+^ ions have magnetic moments with the following values: 5, 2 and 5 μ_B_ respectively [20]. The distribution of cations between tetrahedral (A) and octahedral (B) sites of the spinel decides the magnetic moment per formula unit. The addition of Mn^2+^ ions in the Ni ferrite can induce a migration of the Fe^3+^ ions from the tetrahedral (A) to the octahedral (B) sites leading to a spin imbalance between the two sites, resulting in an increase of the magnetization at the octahedral (B) sites [20]. The surface energy of nanosized particles is large and can modify the typical cation distribution between the A and B sites [3,5]. The SiO_2_ matrix can partially dilute the magnetic matrix of the cations and it can create disorder at the surface of the particles and increase the number of defects, broken bonds, canted spins, and pinning of the magnetic field lines [2,5]. The nanoparticles calcined at 700 °C have rather low values of *M_S_* since they show lower crystallinity, large defect concentration, reduced coordination number and increased interatomic spacing [5]. The *M_S_* values of NCs calcined at 700 °C increase with increasing N_i0_._6_Mn_0.4_Fe_2_O_4_ content, not far from a linear dependence, from 2.5 emu/g (α = 25%) to 31.5 emu/g (α = 100%). This behavior indicates that the main contribution to magnetization is given by the ferrite content in the samples. A possible explanation of the deviation from the linear dependence can be the disorder of magnetic moments on the surface of particles, mainly for the small size particles which have a higher surface-to-volume ratio [2,5]. The increase of *M_S_* with increasing particle sizes is typical for nano-sized ferrites [28]. Excepting the sample with α = 25%, there is a very good proportionality between particle and the crystallite sizes. The crystallite sizes also increase continuously with the ferrite content. This behavior suggests that the SiO_2_ content has a negligible effect on the interaction between the magnetic moments of the cations from tetrahedral (A) and octahedral (B) sites, i.e., the magnetic order is not significantly changed by the SiO_2_ matrix. The *H_C_* decreases with increasing ferrite content, or with growing of the crystallite sizes as expected for multi-domain nanoparticles [28,29]. The *H_C_* decreases from 185 Oe (α = 25%) to 126 Oe (α = 100%). The *M_R_* decreases from 4.5 emu/g (α = 100%) to 0.68 emu/g (α = 25%) mainly due to the increasing disorder of the magnetic moments in the outer shell of the smaller sized particles [2,5]. The magnetic properties of these NCs are also affected by their bulk densities and by their grain sizes and grain size distributions. The strain released by the larger particles is higher than those of the smaller ones, resulting in lattice expansion. The pores can also contribute to the magnetic properties of the NCs, acting as pinning centers for the domain walls and for the magnetic moments of the cations [5]. The observed *M_S_* values are in good agreement with the cation distribution theory and Neel’s molecular field model [1]. The lower values of *M_S_* for some of the NCs can be explained by the effect of the spin canting in the frame of the non-colinear Yafet-Kittel model in the presence of Jahn-Teller cations [8].

The coercive field, *H_C_*, is given mainly by the magneto-crystalline anisotropy, but also by the exchange anisotropy due to the magnetic moment’s interaction from the particle surface [15]. Generally, the M–H curves do not reach complete magnetic saturation, even in 10 T. For these cases, the *M_S_* was estimated by using the law of approach to magnetic saturation [30,31]. The absence of complete saturation in ferromagnetic nanoparticles is generally related to the magnetic moments’ disorder in the surface layers of the particles which needs a larger magnetic field for saturation, in association with the lower anisotropy of the smaller sized particles [10]. The *H_C_* values are rather low, in the range from 126 to 260 Oe. As can be seen, the *M_S_* increases for the NCs with lower SiO_2_ matrix content. This behavior can be related to the decrease of the particle sizes with SiO_2_ content increase and the associated micro-strains, and probably, the magnetic particles morphology and magnetic domain sizes [3]. The *H_C_* decreases nearly linearly with increasing SiO_2_ content due to a continuous decrease of the crystallite sizes in the single-domain range under the influence of the SiO_2_ matrix [9]. The larger sized nanoparticles are composed of multi-domains, where the *H_C_* decreases due to the formation of domain walls in the nanoparticles [7]. The measured *M_S_* values of our previously reported (Zn_0.6_Mn_0.4_Fe_2_O_4_)_α_(SiO_2_)_100−α_ (α = 100%) NCs [5] are similar to those belonging to the (N_i0_._6_Mn_0.4_Fe_2_O_4_)_α_(SiO_2_)_100−α_ (α = 100%) NCs. The *M_S_* of both systems keeps the same trend, decreasing with increasing SiO_2_ matrix content, which results in decreasing particle sizes. The NCs calcined at 1100 °C from the both series behave similarly showing the enhancement of the *H_C_* with increasing SiO_2_ matrix content, in spite of the much larger values of *H_C_* for the Zn_0.6_Mn_0.4_Fe_2_O_4_)_α_(SiO_2_)_100−α_ nanoparticles. These behaviors are typical for particle sizes belonging to the multi-domain range [5,28,29]. The Ni-Mn ferrites calcined at 700 °C also belong to this category, while the previous Zn-Mn ferrites calcined at 700 °C (with smaller particle sizes) behave differently, with a *H_C_* which depreciates with decreasing SiO_2_ matrix content (or with increasing particle sizes), suggesting that most of the particles have sizes belonging to single-domain range.

The obtained Ni_0.6_Mn_0.4_Fe_2_O_4_)_α_(SiO_2_)_100__−α_ NCs belong to an important group of materials with potential for technical application in many biomedical and industrial fields such as drug delivery [30], hyperthermia and healthcare treatment [31,32], biocompatible magnetic fluids [33], magnetic resonance imaging contrast enhancement [34], magnetic data recording [35], microwave applications [36], supercapacitors [37] since these nanoparticles (being passivated) have low toxicity and can be operated by magnetic and electric fields [38,39].

## 4. Conclusions

Sol-gel route followed by calcination was used to synthesize (Ni_0.6_Mn_0.4_Fe_2_O_4_)_α_(SiO_2_)_100−α_ (α = 0, 25, 50, 75, 100%) NCs. In the absence of an SiO_2_ matrix (α = 100%), single-phase crystalline Ni_0.6_Mn_0.4_Fe_2_O_4_ was obtained at 300 °C, while at 700 and 1100 °C, ferrite is accompanied by an α-Fe_2_O_3_ secondary phase. By embedding high ferrite contents in the SiO_2_ matrix (α = 75%), a single phase of Ni_0.6_Mn_0.4_Fe_2_O_4_ was obtained at 300 and 700 °C, but at 1100 °C, besides the crystalline ferrite, α-Fe_2_O_3_ is also present. By embedding the ferrite in equal content with the SiO_2_ matrix (α = 50%), poorly crystallized single-phase Ni_0.6_Mn_0.4_Fe_2_O_4_ is formed at 300 °C, α-Fe_2_O_3_ and Fe_2_SiO_4_ secondary phases accompany the Ni_0.6_Mn_0.4_Fe_2_O_4_ at 700 °C, while at 1100 °C Ni_0.6_Mn_0.4_Fe_2_O_4_ is accompanied by quartz and cristobalite. The embedding of low ferrite content (α = 25%) in the SiO_2_ matrix results in similar crystalline phases as in the case of NCs with α = 50% except that Fe_2_SiO_4_ secondary phase is also formed at 1100 °C. The increase of the calcination temperature and ferrite content embedded in the SiO_2_ matrix led to an increase of the average crystallites size: 2.6–4.6 nm (300 °C), 16.5–50.1 nm (700 °C) and 30.3–74.5 nm (1100 °C). AFM investigation revealed that the average particle diameter increases with increasing calcination temperature, while the amorphous SiO_2_ acts as an insulator among magnetic crystallites and prevents their overgrowth, especially at 1100 °C. The magnetic parameters enhance with increasing Ni_0.6_Mn_0.4_Fe_2_O_4_ content embedded in the SiO_2_ matrix: *M_S_* from 2.5 to 31.5 emu/g (700 °C) and from 4.5 to 80.5 emu/g (1100 °C), *M_R_* from 0.68 to 4.5 emu/g (700 °C) and from 1.1 to 12.6 emu/g (1100 °C), *H_C_* from 126 to 186 Oe (700 °C) and from 150 to 260 Oe (1100 °C). The embedding of ferrite in the SiO_2_ matrix led to the particle sizes decreasing in the nano-range, but also to the alteration of the magnetic parameters. As expected, unembedded Ni_0.6_Mn_0.4_Fe_2_O_4_ (α = 100%) is ferromagnetic, the SiO_2_ matrix (α = 0%) is diamagnetic with a small ferromagnetic fraction, while the Ni_0.6_Mn_0.4_Fe_2_O_4_ embedded in SiO_2_ is superparamagnetic. The obtained NCs can be further developed to obtain soft and thin magnetic films on various solid substrates with tailored properties by varying the ferrite-to-matrix ratio and by a proper management of adsorption process.

## Figures and Tables

**Table 1:** Structural parameters of (N_i0_._6_Mn_0.4_Fe_2_O_4_)_α_(SiO_2_)_100−α_ NCs calculated from AFM and XRD data.

α, %	Calcination Temperature, °C	Roughness, nm	Average Particle Diameter, nm	Average Crystallite Size, nm	Crystallinity, %
100	300	1.0 ± 0.2	18 ± 2	4.6 ± 0.3	14 ± 1
	700	0.8 ± 0.2	52 ± 3	50 ± 3	81 ± 5
	1100	2.3 ± 0.6	75 ± 4	75 ± 5	98 ± 6
75	300	1.4 ± 0.4	20 ± 5	3.8 ± 0.3	12 ± 1
	700	1.1 ± 0.3	35 ± 3	28 ± 2	42 ± 3
	1100	2.9 ± 1.0	58 ± 5	44 ± 3	72 ± 4
50	300	1.1 ± 0.3	14 ± 1	2.6 ± 0.2	8.0 ± 0.5
	700	0.9 ± 0.2	28 ± 4	19 ± 1	25 ± 2
	1100	1.1 ± 0.4	52 ± 5	38 ± 2	66 ± 4
25	300	0.8 ± 0.2	16 ± 2	-	amorphous
	700	1.0 ± 0.3	30 ± 4	17 ± 1	21 ± 1
	1100	1.3 ± 0.3	48 ± 4	30 ± 2	56 ± 3
0	300	0.9 ± 0.2	12 ± 3	-	amorphous
	800	2.0 ± 0.8	28 ± 3	-	amorphous
	1100	2.2 ± 0.8	35 ± 4	-	amorphous

## Data Availability

Data are available from the corresponding author upon request.

## References

[1] Mathubala G., Manikandan A., Arul Antony S., Ramar P. (2016). Photocatalytic degradation of methylene blue dye and magnetooptical studies of magnetically recyclable spinel Ni_x_Mn_1-x_Fe_2_O_4_ (x = 0.0-1.0) nanoparticles. J. Molec. Struct..

[2] Atif M., Sato Turtelli R., Grössinger R., Siddique M., Nadeem M. (2014). Effect of Mn substitution on the cation distribution and temperature dependence of magnetic anisotropy constant in Co_1-x_Mn_x_Fe_2_O_4_ (0.0 ≤ x ≤ 0.4) ferrites. Ceram. Int..

[3] Suresh J., Trinadh B., Babu B.V., Reddy P.V.S.S.S.N., Mohan B.S., Krishna A.R., Samatha K. (2021). Evaluation of micro-structural and magnetic properties of nickel nano-ferrite and Mn^2+^ substituted nickel nano-ferrite. Phys. B Cond. Matter..

[4] Airimioaei M., Ciomaga C.E., Apostolescu A., Leonite L., Iordan A.R., Mitoseriu L., Palamaru M.N. (2011). Synthesis and functional properties of the Ni_1−x_Mn_x_Fe_2_O_4_ ferrites. J. Alloys Comp..

[5] Dippong T., Deac I.G., Cadar O., Levei E.A. (2021). Effect of silica embedding on the structure, morphology and magnetic behavior of (Zn_0.6_Mn_0.4_Fe_2_O_4_)_δ_/(SiO_2_)_(100-δ)_ nanoparticles. Nanomaterials.

[6] Marinca T.F., Chicinaș I., Isnard O., Neamțu B.V. (2016). Nanocrystalline/nanosized manganese substituted nickel ferrites—Ni_1-x_Mn_x_Fe_2_O_4_ obtained by ceramic-mechanical milling route. Ceram. Int..

[7] Maaz K., Duan J.L., Karim S., Chen Y.H., Zhai P.F., Xu L.J., Yao H.J., Liu J. (2016). Fabrication and size dependent magnetic studies of Ni_x_Mn_1-x_Fe_2_O_4_ (x = 0.2) cubic nanoplates. J. Alloys Comp..

[8] Abdallah H.M.I., Moyo T. (2014). Superparamagnetic behavior of Mn_x_Ni_1-x_Fe_2_O_4_ spinel nanoferrites. J. Magn. Mater..

[9] Shobana M.K., Sankar S. (2009). Structural, thermal and magnetic properties of Ni_1-x_Mn_x_Fe_2_O_4_ nanoferrites. J. Magn. Mater..

[10] Dippong T., Levei E.A., Cadar O. (2021). Recent advances in synthesis and applications of MFe_2_O_4_ (M = Co, Cu, Mn, Ni, Zn) nanoparticles. Nanomaterials.

[11] Dippong T., Deac I.G., Cadar O., Levei E.A., Petean I. (2020). Impact of Cu^2+^ substitution by Co^2+^ on the structural and magnetic properties of CuFe_2_O_4_ synthesized by sol-gel route. Mater. Caract..

[12] Swarthmore P. (1999). Powder Diffraction File, Joint Committee on Powder Diffraction Standards.

[13] Jesudoss S.K., Judith Vijaya J., John Kennedy L., Iyyappa Rajana P., Al-Lohedan A.H., Jothi Ramalingam R., Kaviyarasu K., Bououdina M. (2016). Studies on the efficient dual performance of Mn_1−x_Ni_x_Fe_2_O_4_ spinel nanoparticles in photodegradation and antibacterial activity. J. Photochem. Photobiol. B Biol..

[14] Machala L., Tucek J., Zboril R. (2011). Polymorphous transformations of nanometric iron(III) oxide: A review. Chem. Mater..

[15] Shahmoradi Y., Souri D. (2019). Growth of silver nanoparticles within the tellurovanadate amorphous matrix: Optical band gap and band tailing properties, beside the Williamson-Hall estimation of crystallite size and lattice strain. Ceram. Int..

[16] Auderbrand N., Auffredic J.P., Louer D. (1998). X-ray diffraction study of the early stages of the growth of nanoscale zinc oxide crystallites obtained from thermal decomposition of four precursors. General concepts on precursor-dependent microstructural properties. Chem. Mater..

[17] Köseoğlu Y. (2013). Structural, magnetic, electrical and dielectric properties of Mn_x_Ni_1-x_Fe_2_O_4_ spinel nanoferrites prepared by PEG assisted hydrothermal method. Ceram Int..

[18] Minakshi M.S., Watcharatharapong T., Chakraborty S., Ahuja R., Duraisamy S., Rao P.T., Munichandraiah N. (2015). Synthesis, and crystal and electronic structure of sodium metal phosphate for use as a hybrid capacitor in non-aqueous electrolyte. Dalton Trans..

[19] Minakshi M., Sharma N., Ralph D., Appadoo D., Nallathamby K. (2011). Synthesis and characterization of Li(Co_0.5_Ni_0.5_)PO_4_ cathode for Li-Ion aqueous battery applications. Electrochem. Solid State Lett..

[20] Hussain A., Abbas T., Niazi S.B. (2013). Preparation of Ni_1−x_Mn_x_Fe_2_O_4_ ferrites by sol-gel method and study of their cation distribution. Ceram Int..

[21] Al-Hada N.M., Kamari H.M., Shaari A.H., Saion E. (2019). Fabrication and characterization of manganese-zinc ferrite nanoparticles produced utilizing heat treatment technique. Res. Phys..

[22] Ashiq N.M., Ehsan M.F., Iqbal M.J., Gul I.H. (2011). Synthesis, structural and electrical characterization of Sb^3+^ substituted spinel nickel ferrite (NiSb_x_Fe_2−x_O_4_) nanoparticles by reverse micelle technique. J. Alloys Comp..

[23] Tong S.-K., Chi P.-W., Kung S.H., Wei D.H. (2018). Tuning bandgap and surface wettability of NiFe_2_O_4_ driven by phase transition. Sci. Rep..

[24] Enuka E., Monne M.A., Lan X., Gambin V., Koltun R., Chen M.Y. (2020). 3D inkjet printing of ferrite nanomaterial thin films for magneto-optical devices. Quantum Sensing and Nano Electronics and Photonics, Proceedings of the Photonic West 2020, San Francisco, CA, USA, 31 January 2020.

[25] Uda M.N.A., Gopinath S.C.B., Hashim U., Halim N.H., Parmin N.A., Afnan Uda M.N., Anbu P. (2020). Production and characterization of silica nanoparticles from fly ash: Conversion of agro-waste into resource. Prep. Biochem. Biotechnol..

[26] Schaeffer D.A., Polizos G., Smith D.B., Lee D.F., Hunter S.R., Datskos P.G. (2015). Optically transparent and environmentally durable superhydrophobic coating based on functionalized SiO_2_ nanoparticles. Nanotechnology.

[27] Minakshi M. (2010). Lithium intercalation into amorphous FePO_4_ cathode in aqueous solutions. Electrochim. Acta.

[28] Li Q., Kartikowati C.W., Horie S., Ogi T., Iwaki T., Okuyama K. (2017). Correlation between particle size domain structure and magnetic properties of highly crystalline Fe_3_O_4_ nanoparticles. Sci. Rep..

[29] Cullity B.D., Graham C.D. (2011). Introduction to Magnetic Materials.

[30] Devi I.E., Soibam C. (2019). Law of approach to saturation in Mn-Zn ferrite nanoparticles. J. Supercond. Nov. Magn..

[31] Brown W.F. (1940). Theory of the approach to magnetic saturation. Phys. Rev..

[32] Mondalek F.G., Zhang Y.Y., Kropp B., Kopke R.D., Ge X., Jackson R.L., Dormer K.J. (2006). The permeability of SPION over an artificial three-layer membrane is enhanced by external magnetic field. J. Nanobiotechnology.

[33] Pankhurst Q.A., Connolly J., Jones S.K., Dobson J. (2003). Applications of magnetic nanoparticles in biomedicine. J. Phys. D Appl. Phys..

[34] Umut E., Coşkun M., Pineider F., Berti D., Güngüneş H. (2019). Nickel ferrite nanoparticles for simultaneous use in magnetic resonance imaging and magnetic fluid hyperthermia. J. Colloid Interface Sci..

[35] Bhardwaj A., Parekh K., Jain N. (2020). In vitro hyperthermic effect of magnetic fluid on cervical and breast cancer cells. Sci. Rep..

[36] Huh Y.M., Jun Y.W., Song H.T., Kim S., Choi J.S., Lee J.H., Yoon S., Kim K.S., Shin J.S., Suh J.S. (2005). In vivo magnetic resonance detection of cancer by using multifunctional magnetic nanocrystals. J. Am. Chem. Soc..

[37] Chakradharya V.K., Ansaria A., Akhtara M.J. (2019). Design, synthesis, and testing of high coercivity cobalt doped nickel ferrite nanoparticles for magnetic applications. J. Magn. Mater..

[38] Pardavi-Horvath M.J. (2000). Microwave applications of soft ferrites. J. Magn. Mater..

[39] Sharif S., Yazdani A., Rahimi K. (2020). Incremental substitution of Ni with Mn in NiFe_2_O_4_ to largely enhance its supercapacitance properties. Sci. Rep..

